# Giant ascending aortic pseudoaneurysm requiring third re-do sternotomy more than two decades after aortic root replacement in an octogenarian: a case report

**DOI:** 10.1093/jscr/rjag809

**Published:** 2026-09-16

**Authors:** Quincy C Guenther, Thomas Coleman, Christopher T Salerno, Miroslav P Peev

**Affiliations:** Department of Surgery, Section of Cardiac Surgery, University of Chicago Medicine, Chicago, IL, United States; Department of Surgery, Section of Cardiac Surgery, University of Chicago Medicine, Chicago, IL, United States; Department of Surgery, Section of Cardiac Surgery, University of Chicago Medicine, Chicago, IL, United States; Department of Surgery, Section of Cardiac Surgery, University of Chicago Medicine, Chicago, IL, United States

**Keywords:** ascending aortic pseudoaneurysm, redo sternotomy, extended hemiarch replacement, antegrade cerebral perfusion

## Abstract

Ascending aortic pseudoaneurysm (AAP) is a rare but potentially fatal late complication of cardiac and aortic surgery. Surgical repair becomes particularly challenging when the lesion is large, adherent to the posterior table of the sternum, and encountered during repeat sternotomy. We report the successful management of an 11-cm AAP arising from distal graft dehiscence more than two decades after mechanical aortic root replacement in an 82-year-old man. Owing to the high risk of catastrophic haemorrhage during re-entry, peripheral cardiopulmonary bypass, profound hypothermia was established before sternotomy. The pseudoaneurysm was successfully resected and reconstructed with extended hemiarch replacement. The patient experienced an uncomplicated recovery and was discharged home on postoperative day seven. This case highlights key technical considerations in the repair of complex ascending aortic pseudoaneurysms.

## Introduction

Ascending aortic pseudoaneurysm (AAP) is an uncommon but serious complication following cardiac and aortic surgery, with a reported incidence of ~0.5% [1]. Most lesions originate from anastomoses, cannulation and vent sites, or infection [2, 3]. Unlike true aneurysms, pseudoaneurysms lack a complete vessel wall and carry a substantial risk of rupture and death. Mortality rates exceeding 60% have been reported in untreated patients [4].

Although surgical repair remains the standard treatment, operative management is frequently challenging. Re-entry through a previous sternotomy may precipitate catastrophic hemorrhage, particularly when the pseudoaneurysm occupies the retrosternal space.

We report repair of a giant AAP caused by distal graft dehiscence >20 years after mechanical aortic root replacement in an octogenarian patient. This case highlights key technical considerations in complex reoperative aortic surgery.

## Case report

An 82-year-old man with a history of two prior median sternotomies was transferred emergently to our institution for evaluation of a large ascending aortic pseudoaneurysm. He previously underwent aortic valve replacement followed by mechanical composite root replacement 20 years before presentation.

**Figure 1:**
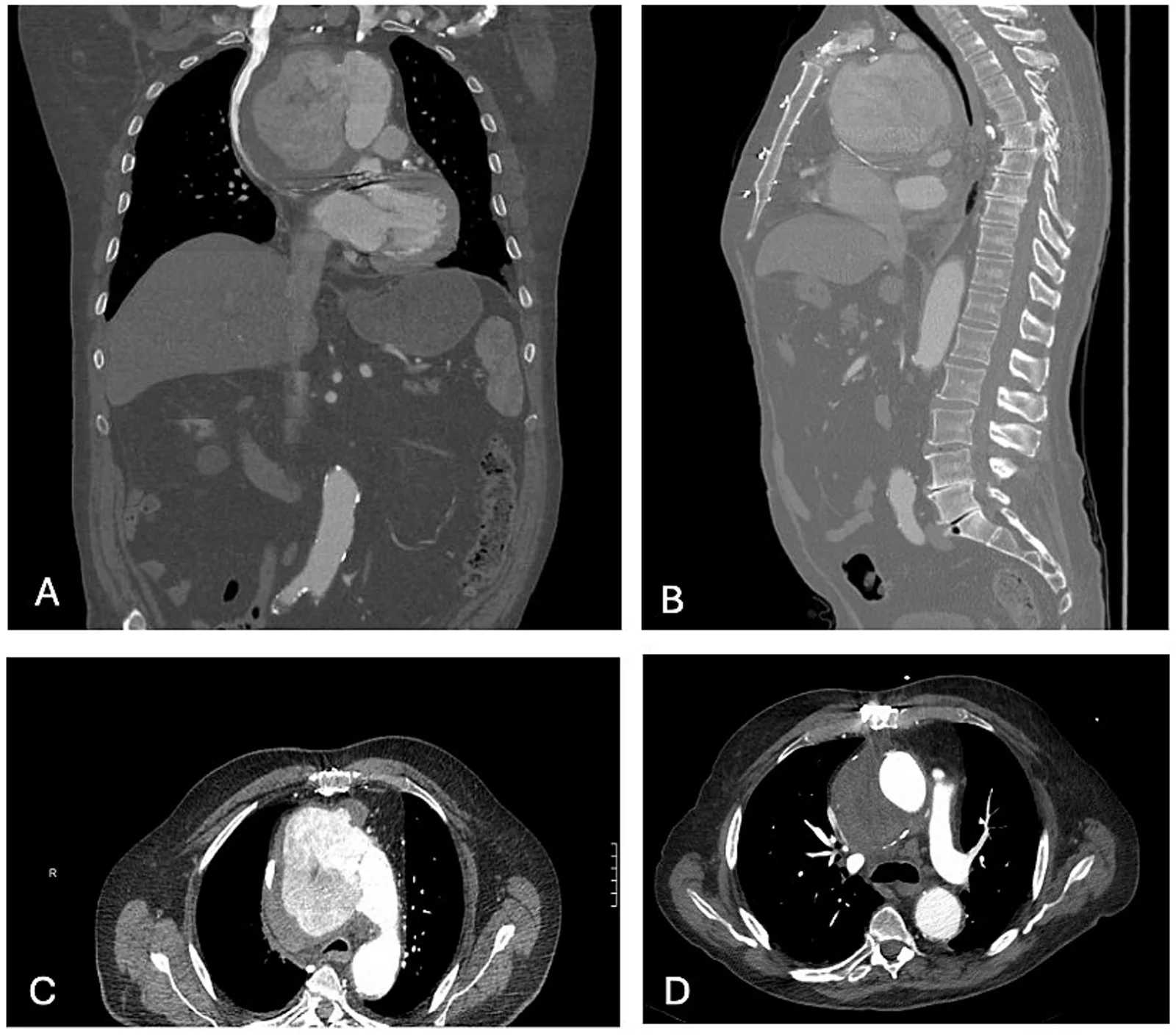
Preoperative CTA chest demonstrates massive pseudoaneurysm with compression of the superior vena cava, the main pulmonary artery and significant displacement of the innominate and left common carotid arteries. The wall of the pseudoaneurysm is adherent to the posterior sternal table (A-C). Postoperative CTA demonstrates the size of the ascending graft in comparison with the residual pseudoaneurysm cavity (D).

The patient initially presented to an outside hospital with progressive chest pain and shortness of breath. Computed tomography angiography (CTA) demonstrated an 11-cm AAP arising from the distal graft anastomosis (Fig. 1). The lesion occupied the anterior mediastinum and was deeply adherent to the posterior sternum.

Despite the radiographic findings, the patient remained functionally independent with minimal frailty. Urgent operative repair was recommended because of rupture risk.

### Operative technique

Given the intimate relationship between the pseudoaneurysm and the sternum, direct re-entry was considered prohibitively hazardous. The right common femoral artery and vein were exposed through a groin cutdown and cannulated following systemic heparinization.

Cardiopulmonary bypass was initiated and systemic cooling commenced to a target bladder temperature of 18°C. Once the target temperature was achieved, a third sternotomy was performed while being prepared to initiate hypothermic circulatory arrest if the pseudoaneurysm was entered. Entry into the mediastinum revealed severe distortion of normal anatomy by the giant pseudoaneurysm. The innominate artery was carefully dissected and cannulated with a 12 Fr pediatric arterial cannula. Following snaring of the vessel origin, unilateral antegrade cerebral perfusion was initiated at 12 ml/kg/min.

**Figure 2:**
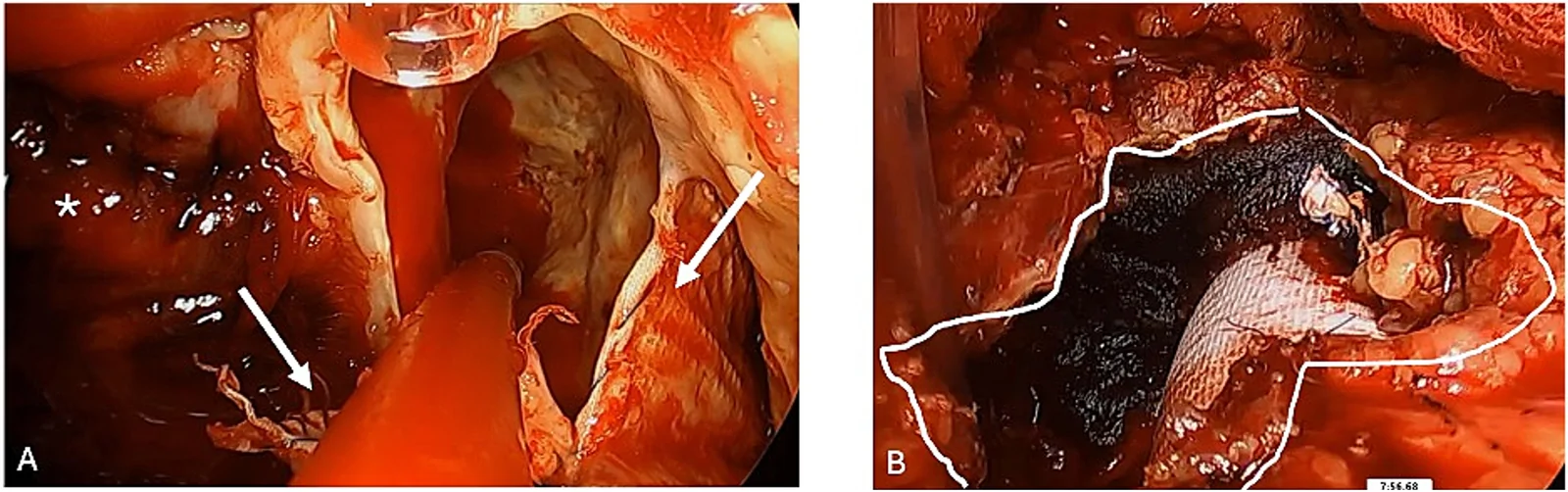
Intraoperative images: (A) large pseudoaneurysm cavity (*) and more than 180° dehiscence of the previous distal anastomosis suture line; (B) demonstration of the new (extended hemiarch) surgical graft within the massive pseudoaneurysm cavity.

The pseudoaneurysm cavity was opened. The heart was arrested with the help of direct ostial Del Nido cardioplegia. Inspection revealed ~180° of distal graft dehiscence (Fig. 2A).

The disrupted anastomosis was excised and the distal aorta reconstructed in an extended hemiarch fashion. A 32-mm Dacron chimney graft was sewn to the reconstructed arch. Systemic perfusion was restored and antegrade cerebral perfusion discontinued.

The proximal reconstruction was completed with a graft-to-graft anastomosis to the existing mechanical Bentall conduit (Fig. 2B). The patient was weaned from cardiopulmonary bypass with minimal pharmacologic support.

### Postoperative course

The patient was extubated 6 hours postoperatively with intact neurologic function. Recovery progressed without major complications, and he was discharged home on postoperative Day 7.

Follow-up computed tomography at 1, 3, and 6 months demonstrated an intact repair without recurrent pseudoaneurysm or anastomotic complications. At latest follow-up, the patient had returned to his baseline functional status.

## Discussion

AAP remains a rare but potentially lethal late complication of previous cardiac surgery. This case is notable for its size, third redo sternotomy, and successful repair in an octogenarian.

The delayed presentation occurring >20 years after root replacement underscores the lifelong risk of anastomotic degeneration following complex aortic reconstruction and highlights the importance of maintaining a high index of suspicion in patients with previous aortic surgery who present with new cardiopulmonary symptoms.

Although transcatheter solutions have been reported for selected ascending aortic pseudoaneurysms [5, 6], the lesion’s size, broad neck, and proximity to the arch made open repair the preferred treatment [7].

The greatest technical challenge was safe mediastinal re-entry. Establishing peripheral cardiopulmonary bypass before sternotomy allowed systemic cooling and immediate access to circulatory arrest if rupture occurred, a well-established strategy in complex reoperative aortic surgery [4].

Selection of cerebral protection and arterial inflow strategies also required careful consideration. Direct carotid and axillary artery cannulation are established options for cerebral protection during aortic arch reconstruction [8]. Carotid atherosclerosis and small-caliber axillary vessels limited alternative cannulation strategies. Innominate cannulation provided reliable unilateral antegrade cerebral perfusion.

This case also highlights the importance of physiologic rather than chronologic age when evaluating candidates for complex aortic surgery. Despite being 82 years old and undergoing a third sternotomy, the patient recovered well, supporting consideration for surgery in carefully selected elderly patients.

## Conclusion

Giant AAP following prior aortic root replacement presents unique technical challenges. Successful management requires careful preoperative planning and cerebral protection strategies. This case demonstrates safe repair of a giant pseudoaneurysm requiring third redo sternotomy in a carefully selected octogenarian.
